# Surgical Intervention in Pediatric Orbital Hemangioma: A Case Report

**DOI:** 10.7759/cureus.50156

**Published:** 2023-12-08

**Authors:** Mazen Almatrudi, Khalaf Alnowaishiri, Sabrin Babiker, Demah Alsufyani, Ahmed Abdelaziz

**Affiliations:** 1 General Practice, Qassim University, Qassim, SAU; 2 General Practice, University of Jordan, Amman, JOR; 3 General Practice, National Ribat University, Khartoum, SDN; 4 General Practice, Taif University, Taif, SAU; 5 Ophthalmology, Dallah Hospital, Riyadh, SAU

**Keywords:** vascular lesion, infantile hemangioma, propranolol, proptosis, orbital hemangioma

## Abstract

Infantile hemangiomas are common vascular tumors in infancy, typically affecting the skin. However, intraconal orbital hemangiomas are rare and present diagnostic challenges due to their impact on critical structures within the orbit. We present the case of a 1-year-old male with progressive orbital swelling and proptosis. Initial conservative management with propranolol was attempted, but due to the lack of response, surgical resection of the intraconal hemangioma was performed. Pediatric orbital hemangiomas, particularly intraconal variants, pose distinctive diagnostic challenges. While conservative approaches, such as propranolol, align with evolving strategies for infantile hemangiomas, surgical intervention may be necessary in cases with an inadequate response. This case underscores the importance of recognizing the need for timely surgical intervention in pediatric orbital hemangiomas, even after initial conservative measures. Successful surgical outcomes contribute to understanding pediatric orbital pathology and emphasize the ongoing evolution of management strategies in this challenging clinical scenario.

## Introduction

Infantile hemangiomas, the most prevalent vascular tumors in infancy, typically manifest within the first few weeks of life, exhibiting a distinct growth phase followed by spontaneous involution [1]. These hemangiomas arise from abnormal vascular development during embryogenesis, involving the proliferation of endothelial cells and subsequent vascular remodeling [2].

Vascular anomalies within the orbit and orbital hemangiomas can impact various compartments, including the intraconal space region deep within the orbit housing critical structures like the optic nerve and extraocular muscles [1,2]. While these lesions commonly affect the skin, their occurrence within the orbit, particularly in the intraconal space - housing critical structures such as the optic nerve and extraocular muscles - is a rare phenomenon [1]. Epidemiologically, infantile hemangiomas have been reported to occur in approximately 4-5% of infants, making them a relatively common vascular tumor in this age group [2,3].

In this report, we present a case of a 1-year-old male with a rare intraconal orbital hemangioma, emphasizing the diagnostic challenges associated with such cases. Despite conservative measures, the lack of response prompted a shift to surgical resection. This case contributes to the understanding of pediatric orbital lesions.

## Case presentation

We present the case of a 1-year-old male brought to our attention due to parental concern regarding their child's progressive orbital swelling and proptosis. The parents noticed a subtle swelling near the right eye three months before the consultation, which gradually increased in size. The patient had an unremarkable antenatal and perinatal history, and developmental milestones were age-appropriate.

Upon physical examination, the child appeared well-nourished and in no acute distress. Mild proptosis of the right eye with associated periorbital edema was noted. Extraocular movements were limited, particularly in upward and lateral gaze. Palpation revealed a firm consistency of the globe, with no tenderness or warmth. Visual acuity and pupillary reactions were within normal limits. Physical examination of other systems was unremarkable.

**Figure 1:**
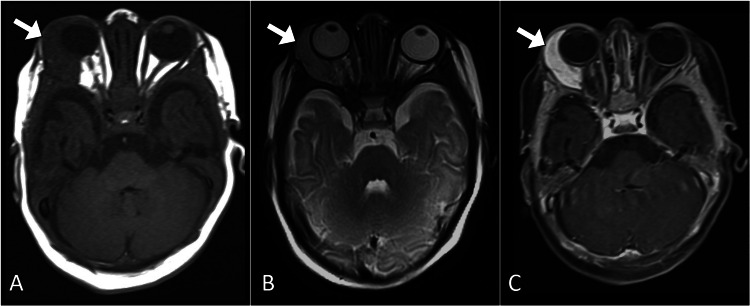
Axial MR images of the brain at the orbital level. The images illustrate a right intraconal orbital lesion (arrow) with distinctive features: low signal intensity on the T1-weighted image (A), high signal intensity on the T2-weighted image (B), and avid post-contrast enhancement (C), confirming its vascular nature. MR: magnetic resonance

Initial laboratory investigations, including a complete blood count and basic metabolic panel, showed no abnormalities. The coagulation profile was normal. A broad differential diagnosis was considered, including vascular malformations, hemangiomas, and rare neoplasms. Imaging studies were crucial for further evaluation. Magnetic resonance imaging of the orbit revealed an intraconal lesion of the right orbit that exhibited low signal intensity on T1-weighted images, high signal intensity on T2-weighted images, and avid enhancement on post-contrast images. The lesion was associated with mild proptosis (Figure 1).

Given the patient's age and the generally benign nature of the lesion, a multidisciplinary team comprising pediatricians, pediatric ophthalmologists, and interventional radiologists collaboratively opted for an initial conservative management approach. This approach involved the administration of propranolol. The conservative management plan extended over six months, with regular assessments during this period aimed at monitoring the response to propranolol and assessing any changes in the lesion's characteristics.

Despite the extended duration of conservative treatment, the patient exhibited a lack of response, and there was a noticeable progression of symptoms, indicating an insufficient response to propranolol and the need for surgical management. The surgical resection of the intraconal hemangioma was carried out by a pediatric ophthalmologist. Postoperatively, propranolol was continued as part of the management strategy to prevent recurrence and optimize the long-term outcome. The histopathological examination of the resected lesion confirmed the diagnosis of cavernous hemangioma, providing crucial insights into the nature of the lesion and guiding postoperative management (Figure 2).

**Figure 2:**
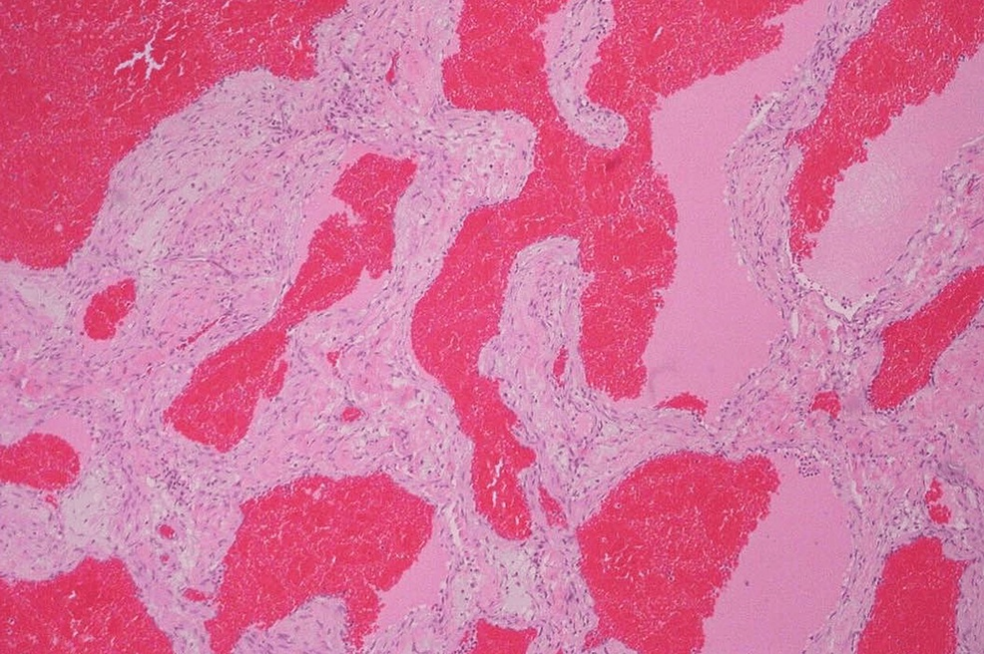
2. Histopathological image reveals cavernous vascular spaces filled with blood, consistent with a cavernous hemangioma.

Throughout the hospital course, the patient demonstrated a favorable response to the combined surgical and medical approach, with improved extraocular movements. The child was discharged with a scheduled follow-up plan to monitor for any recurrence or complications.

During the follow-up period, the patient continued to show sustained improvement. Long-term follow-up is planned to assess the need for ongoing therapy and to monitor for any potential late sequelae.

## Discussion

Cavernous hemangiomas of the orbit, recognized as the most common benign vascular tumor of the infant orbit, typically present as a slow, progressive intraorbital mass, often leading to late exophthalmos [1]. Clinical features include reduced visual acuity, ocular motility disorders, and retro-orbital pain. Predominantly affecting the middle third of the orbit, cavernous hemangiomas tend to favor the intraconal space, resulting in progressive axial proptosis.

Imaging studies, including ultrasound, computed tomography, and magnetic resonance imaging, play a pivotal role in diagnosing cavernous hemangiomas of the orbit. Distinctive imaging features encompass a well-defined, encapsulated lesion with hyperdensity on computed tomography. On magnetic resonance imaging, the lesion typically exhibits iso-intensity or slight hypo-intensity on T1-weighted sequences and hyperintensity on T2-weighted sequences, with characteristic contrast enhancement [1,3].

Treatment modalities for cavernous hemangiomas of the orbit typically involve surgery, particularly when the lesion induces clinical manifestations. Surgical approaches vary based on the lesion's location within the orbit. Lesions in the superomedial aspect may necessitate a transcranial route, while retrobulbar intraconal lesions, especially those anteriorly located, may be approached through a lateral orbitotomy [2,4]. In our pediatric case, after the failure of the initial conservative management with propranolol, surgical intervention was pursued with a lateral orbitotomy, chosen due to the location of the hemangioma between the optic nerve and extraocular muscles.

In our pediatric case, the initial attempt at conservative management with propranolol aligns with evolving strategies in the management of infantile hemangiomas. However, the lack of response necessitated surgical intervention, paralleling the infantile cavernous hemangiomas of the orbit case.

Surgical outcomes in cavernous hemangiomas of the orbit are generally favorable, carrying a good vital and functional prognosis. However, recognized risks include damage to the optic nerve leading to blindness. Careful surgical dissection is crucial, impacting ocular motility and the potential for postoperative visual impairment [2,5].

## Conclusions

In conclusion, this case underscores key considerations in the diagnosis and management of pediatric orbital hemangiomas, especially those within the intraconal space. Although conservative approaches, such as propranolol, are commonly employed for infantile hemangiomas, our case emphasizes the significance of recognizing situations that necessitate a timely shift to surgical intervention. The subsequent successful surgical resection yielded favorable outcomes, contributing to the broader understanding of pediatric orbital pathology.
